# Spinopelvic mobility is influenced by pre-existing contralateral hip arthroplasty: a matched-pair analysis in patients undergoing hip replacement

**DOI:** 10.1186/s13018-022-02945-5

**Published:** 2022-02-02

**Authors:** Maximilian Muellner, Luis Becker, Zhen Wang, Zhouyang Hu, Sebastian Hardt, Matthias Pumberger, Henryk Haffer

**Affiliations:** Center for Musculoskeletal Surgery, Charité - Universitätsmedizin Berlin, corporate member of Freie Universität Berlin, Humboldt-Universität zu Berlin, and Berlin Institute of Health, Berlin, Germany, Berlin, Germany

**Keywords:** Total hip replacement, Total hip arthroplasty dislocation, Spinopelvic complex, Spinopelvic function

## Abstract

**Background:**

Spinopelvic mobility gained increased attention as a contributing factor for total hip arthroplasty (THA) instability. However, it is unknown how a pre-existing THA affects spinopelvic mobility. Therefore, a propensity-score-matched analysis of primary THA patients comparing the individual segments of spinopelvic mobility between patients with pre-existing THA and no-existing THA was conducted. Consequently, the study aimed to discuss (1) whether patients with a pre-existing THA have altered spinopelvic mobility compared to the control group and (2) if spinopelvic mobility changes after THA.

**Methods:**

A prospective observational study enrolled 197 elective primary THA patients, including *N* = 44 patients with a pre-existing unilateral THA. Using propensity-score matching adapted for age, sex, and BMI, *N* = 44 patients without a pre-existing THA were determined. The patients received stereoradiography in standing and relaxed sitting position pre- and postoperatively. Assessed parameters were lumbar lordosis (LL), pelvic tilt (PT), and pelvic femoral angle (PFA). Key parameters of the spinopelvic mobility were defined as lumbar flexibility (∆LL = LL_standing_ − LL_sitting_), pelvic mobility (∆PT = PT_standing_ − PT_sitting_) and hip motion (∆PFA = PFA_standing_ − PFA_sitting_). Pelvic mobility was classified as stiff (∆PT < 10°), normal (∆PT ≥ 10°–30°) and hypermobile (∆PT > 30°). The Wilcoxon rank sum test for dependent samples was used.

**Results:**

Pelvic mobility was significantly increased in the pre-existing THA group (∆PT 18.2° ± 10.7) compared to the control group (∆PT 7.7° ± 8.0; *p* < 0.001) preoperatively and postoperatively (pre-existing: 22.2° ± 9.3; control: 17.0° ± 9.2, *p* = 0.022). Lumbar flexibility was significantly increased in the pre-existing THA group (∆LL 21.6° ± 11.8) compared to the control group (∆LL 12.4° ± 7.8; *p* < 0.001) preoperatively and postoperatively (pre-existing: 25.7° ± 11.0; control: 19.0° ± 10.2; *p* = 0.011). The contribution of stiff pelvic mobility is distinctly smaller in the pre-existing THA group (25%) than in the control group (75%) preoperatively.

**Conclusions:**

Pre-existing THA is associated with significantly enhanced pelvic mobility and lumbar flexibility. Accordingly, we identified the patients without a pre-existing THA as risk candidates with higher likelihood for pathological spinopelvic mobility. This information will assist arthroplasty surgeons in deciding which THA candidates require preoperative radiological screening for pathologic spinopelvic mobility.

**Level of evidence:**

Level II prospective cohort study.

**Supplementary Information:**

The online version contains supplementary material available at 10.1186/s13018-022-02945-5.

## Introduction

Dislocation after total hip arthroplasty (THA) is a severe complication, occurring approximately in 0.2–7% for primary THAs and up to 25% for revision THAs [1]. Several surgeon and implant-associated factors including implant positioning, the surgical approach [1–3], insufficient reconstruction of joint geometry [4–6] and periarticular muscle damage are known to increase the risk for THA dislocation. [1] In addition to the aforementioned risk factors, there are also patient-specific risk factors, for example obesity affecting spinopelvic function [7, 8]. Furthermore, to the previously mentioned aspects, spinopelvic mobility has recently received increased attention by orthopedic surgeons, in the preoperative THA assessment aiming to mitigate the THA patients instability risk [9]. The spinopelvic complex is formed by the lumbar spine, the pelvis, and the hip joint. Accordingly, radiologically assessed key elements of the spinopelvic complex are lumbar lordosis (LL), pelvic tilt (PT) and pelvic femoral angle (PFA). Spinopelvic mobility is defined by the change (∆) of these elements from standing to sitting. Consequently, the three key parameters defining spinopelvic mobility are lumbar flexibility (∆ LL = LL_standing_ − LL_sitting_), pelvic mobility (∆ PT = PT_standing_ − PT_sitting_) and hip motion (∆ PFA = PFA_standing_ − PFA_sitting_) [8, 10].

It is known that decreased lumbar flexibility (∆ LL), increased hip motion (∆ PFA) and stiff pelvic mobility (∆ PT) lead to a significantly increased risk for THA dislocations [11–15]. In addition, spinopelvic hypermobility was also shown to be associated with inferior outcomes and an increased risk of THA dislocations in patients with spinal arthrodesis [16]. Restrictions in any of the elements of the spinopelvic complex are mostly compensated by other segments. This is reflected in patients with lumbar spine degeneration through increased hip motion and pelvic recruitment. Several factors contribute to altered spinopelvic mobility including lumbar fusion and degenerative spine and hip diseases. Studies have demonstrated that osteoarthritis of the hip leads to altered spinopelvic mobility, changes are dependent on the severity of the hip osteoarthritis [17, 18].

However, it is not known if a pre-existing unilateral THA leads to a different spinopelvic mobility in patients receiving a contralateral hip arthroplasty compared to patients undergoing their first hip replacement. Therefore, we performed a propensity score matched analysis using the data of a prospective observational study of patients undergoing THA with a standardized standing and sitting pre- and postoperative EOS assessment comparing the individual elements of spinopelvic mobility (lumbar flexibility, pelvic mobility and hip motion) between patients with pre-existing THA and no-existing THA. Consequently, our study aimed to discuss [1] whether patients with a pre-existing THA have altered spinopelvic mobility compared to the control group and [2] if spinopelvic mobility changes after THA in both assessed groups.

## Materials and methods

A prospective observational radiology study was performed from September 2019 to November 2020 on patients selected for a primary elective THAs at a university hospital. THA was performed by four board certified surgeons. All patients receiving elective THA were screened for study inclusion. Exclusion criteria were defined as bilateral planned THA, severe hip dysplasia with subsequent THA and femur osteotomy, any form of revision THA, any type of fracture such as femoral neck fracture leading to THA, ankylosing spondylitis, spinal fusion surgery at any level, osseous metastasis in the pelvis and neurological pre-existing conditions significantly influencing posture. The study is in compliance with the Helsinki Declaration, has been approved by the institutional ethics board (EA2/142/17) and patients have given their informed written consent.

### Radiographic assessment

**Fig. 1:**
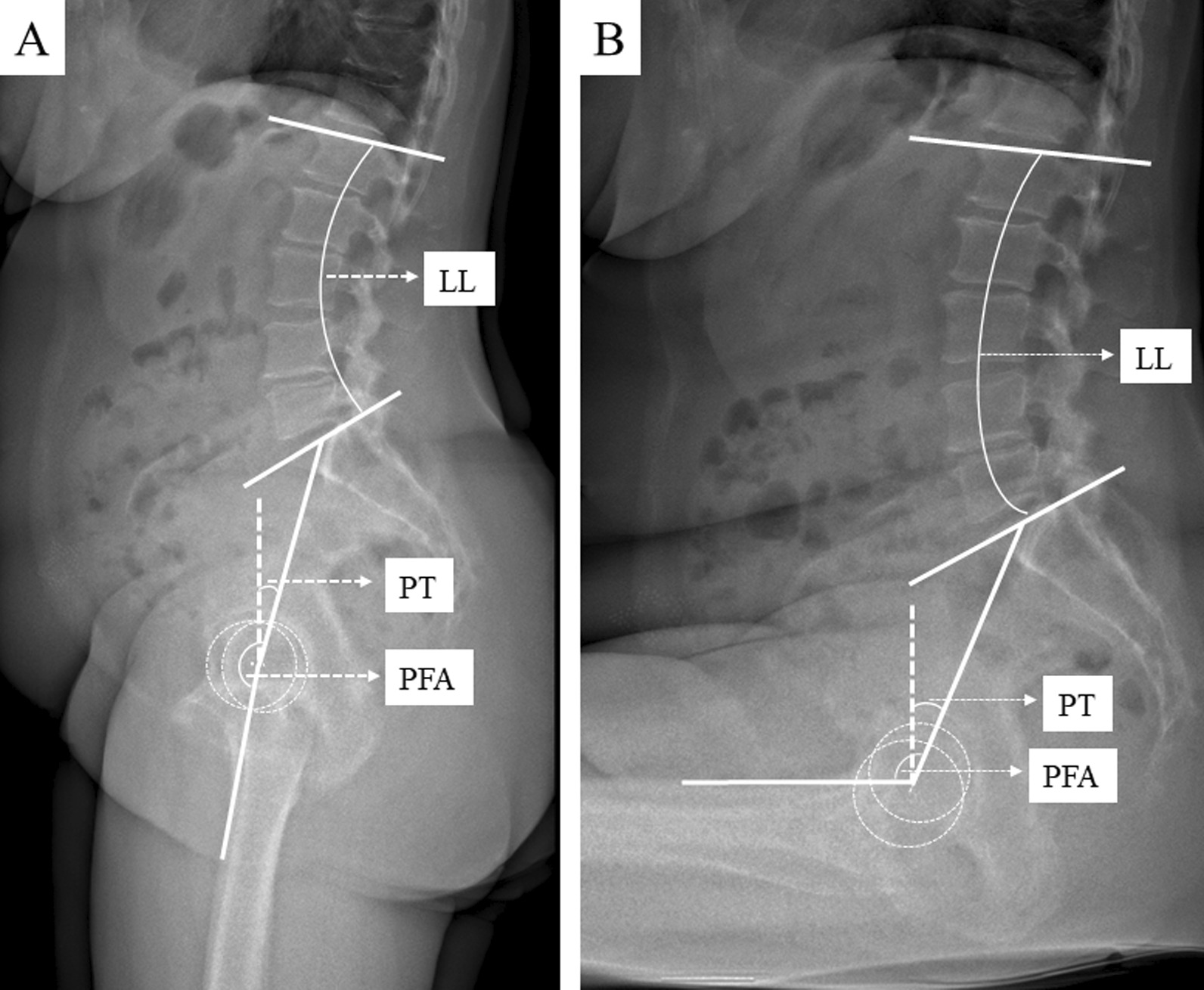
Sagittal standing (A) and sitting (B) EOS radiographs of the spine, pelvis and hip depicting spinopelvic key parameter lumbar lordosis (LL), pelvic tilt (PT) and pelvic femoral angle (PFA)

Within three days pre- and five to seven days postoperatively, the THA patients underwent a complete spine imaging including the pelvis up to the proximal tibia from lateral and anterior posterior in standing and relaxed sitting position using biplanar low-dose stereoradiography (EOS, EOS imaging, Paris, France). The use of the EOS device enables images of the patients performed in functional positions and with a lower radiation dose. Patients were advised to stand naturally, look forward and place their hands on a support with relaxed upper limbs. They were instructed to sit relaxed on a height-adjustable chair without backrest, with the femur parallel to the floor. Radiological measurements were performed by an orthopedic surgeon using Merlin Diagnostic Workcenter (Phoenix PACS, Freiburg, Germany) and randomized 25% of the dataset was measured by a second independent orthopedic surgeon. The spinopelvic parameter lumbar lordosis (LL), pelvic tilt (PT), and pelvic femoral angle (PFA) were measured in both standing and sitting positions and pelvic incidence (PI) in standing position preoperatively and postoperatively (Additional file 1: Table S2, Fig. 1). The differences between the standing and sitting radiographic assessment of LL (∆ LL = LL_standing_ − LL_sitting_), PT (∆ PT = PT_standing_ − PT_sitting_) and PFA (∆ PFA = PFA_standing_ − PFA_sitting_) represent the key elements of spinopelvic mobility [8]. Pelvic mobility determined by ∆ PT < 10° was defined as stiff, ≥ 10–30° as normal and > 30° as hypermobile [19, 20].

### Statistical analyses

All statistical analyses were performed using SPSS Version 27 (IBM Corporation, New York, United States). An age, sex and BMI adapted propensity score matching was conducted, determining the control group with patients without a pre-existing THA before the intervention. The propensity score factor was set to 0.07 for all variables. The Wilcoxon rank sum test for dependent samples was applied in this study. Spearman’s rank correlation coefficient was used to determine the interrater reliability of the radiographic measurements. A significance level of *p* < 0.05 was assumed for all tests.

## Results

**Fig. 2:**
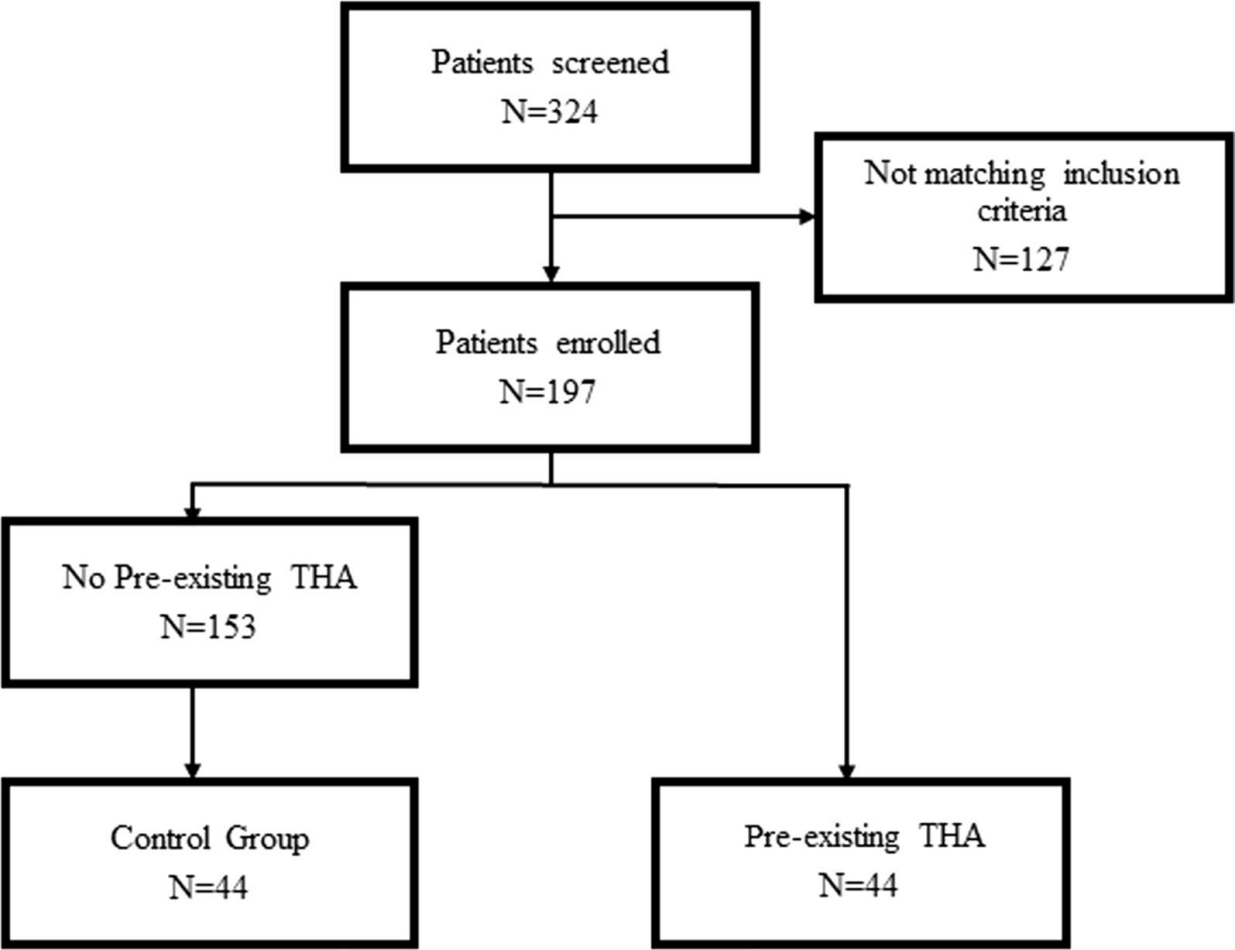
Flowchart of patient inclusion. From the patients enrolled (*N* = 197), *N* = 44 patients were identified with a pre-existing THA, from the remaining *N* = 153 without a pre-existing THA an age, sex and BMI adapted propensity score matching was conducted, determining the control group with patients without a pre-existing THA (*N* = 44)

A total of 324 patients were screened for study eligibility, of those 197 were included and underwent the study protocol with radiographic EOS assessment in standing and sitting position pre- and postoperatively. Of these, 44 had a pre-existing THA. We matched this group for age, sex, and BMI with 44 patients of the remaining 153 patients without a pre-existing THA (Fig. 2). Due to the study design, the groups showed no significant differences according to sex (pre-existing group: 22 females, control group: 22 females; *p* = 1.000), age (pre-existing group: median 71.50, range 31–86; control group: median 70.00, range 31–88; *p* = 0. 353), BMI (pre-existing group: median 27.76 kg/m^2^, range 16.73–51.68 kg/m^2^; control group: median 26.28 kg/m^2^, range 20.31–42.24 kg/m^2^; *p* = 0.172). The radiographic measurements demonstrated adequate interobserver reliability (Additional file 1: Table 1).

**Table 1:** Analyses of spinopelvic complex key elements lumbar flexibility (∆ LL = LL_standing_ − LL_sitting_), pelvic mobility (∆ PT = PT_standing_ − PT_sitting_) and hip motion (∆ PFA = PFA_standing_-PFA_sitting_) and spinopelvic parameter LL, PT and PFA in standing and sitting position of patients with pre-existing THA and the matched control group pre- and postoperatively

Comparison of spinopelvic parameters between pre-existing THA and control group						
	Preoperative			Postoperative		
Parameters	Pre-existing mean (± SD)	Control mean (± SD)	*p* value	Pre-existing mean (± SD)	Control mean (± SD)	*p* value
∆ LL (°)	21.6 (11.8)	12.4 (7.8)	** < .001**	25.7 (11.0)	19.0 (10.2)	**.011**
∆ PT (°)	18.2 (10.7)	7.7 (8.0)	** < .001**	22.2 (9.3)	17.0 (9.2)	**.022**
∆ PFA (°)	55.9 (15.9)	67.9 (14.0)	**.002**	51.1 (11.6)	55.2 (14.0)	.169
LL Stand (°) LL Sit (°)	49.8 (13.6) 28.2 (11.5)	48.8 (15.1) 36.2 (15.6)	.788 **.006**	51.6 (13.4) 25.9 (14.2)	49.7 (14.0) 30.6 (14.4)	.875 .089
PT Stand (°) PT Sit (°)	14.3 (8.1) 32.5 (11.1)	18.4 (8.2) 26.3 (11.2)	**.035** **.021**	12.7 (9.1) 34.9 (10.1)	14.1 (8.7) 31.1 (10.6)	.554 .189
PFA Stand (°) PFA Sit (°)	179.1 (9.8) 123.2 (14.7)	183.9 (11.8) 116.3 (13.4)	**.047** .115	176.5 (10.8) 125.4 (12.7)	178.2 (10.4) 123.2 (15.5)	.612 .464
PI Stand (°)	54.7 (13.1)	56.1 (14.1)	.762	53.8 (12.5)	55.1 (13.0)	.767

*p* values indicating differences between patients with pre-existing THA and the matched control group with no pre-existing THA each preoperatively and postoperatively. Pelvic incidence (PI) is a fixed spinopelvic posture-independent parameter and served as a control parameter revealing no significant differences. Wilcoxon rank sum test was used and level of significance set at *p* < 0.05. SD = standard deviation. Significant differences are marked in bold

### Pre-existing THA and spinopelvic function

**Fig. 3:**
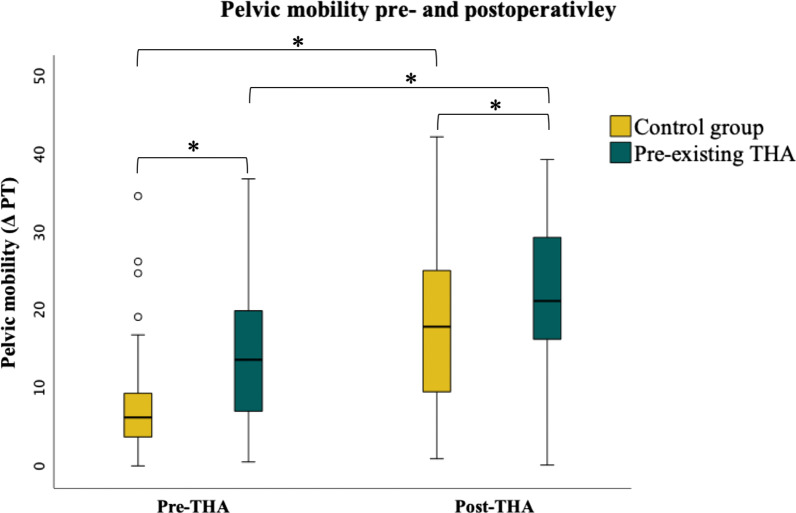
Depicting pelvic mobility based on ∆ PT = PT_standing_-PT_sitting_ according to the pre-existing THA group (green) and the matched control group (yellow) preoperatively and postoperatively. Significant differences are marked by asterisks (*)

Lumbar flexibility (∆ LL) and pelvic mobility (∆ PT) were significantly greater in patients with pre-existing THA compared to the control group preoperatively as well as postoperatively (Fig. 3). Both ∆ LL and ∆ PT elevated due to THA in pre-existing and control group. Hip motion (∆ PFA) was significantly increased preoperatively in the control group compared to the pre-existing THA group. THA resulted in a decrease in ∆ PFA in both groups, resulting in no significant differences between the groups in ∆ PFA after surgery. LL in sitting position preoperatively demonstrated a significant increase in the control compared to pre-existing THA group, while there were no significant differences in LL standing position between the groups. Sitting LL decreased after THA in both groups leading to no significant differences between control and pre-existing THA group postoperatively. Significantly more pelvic retroversion (PT) in standing position and significantly higher sitting PT was observed in the pre-existing THA compared to the control group preoperatively. In both groups, THA led to a reduced pelvic retroversion in standing position and increased sitting pelvic tilt (Table 1).

### Changes of spinopelvic mobility due to THA

Performing THA had significant effects on spinopelvic mobility. In both groups THA resulted in a significant increase in lumbar flexibility and pelvic mobility. There was also a significant reduction in hip motion in both groups after THA (Table 2).

**Table 2:** Analyses of changes in spinopelvic complex elements lumbar flexibility (∆ LL = LL_standing_ − LL_sitting_), pelvic mobility (∆ PT = PT_standing_ − PT_sitting_) and hip motion (∆ PFA = PFA_standing_ − PFA_sitting_) due to THA between preoperative and postoperative

Changes of spinopelvic mobility due to THA				
Parameters	Groups	Preoperative mean (± SD)	Postoperative mean (± SD)	*p* value
∆ LL (°)	Pre-existing	21.6 (11.8)	25.7 (11.0)	**.012**
	Control	12.4 (7.8)	19.0 (10.2)	** < .001**
∆ PT (°)	Pre-existing	18.2 (10.7)	22.2 (9.3)	**.010**
	Control	7.7 (8.0)	17.0 (9.2)	** < .001**
∆ PFA (°)	Pre-existing	55.9 (15.9)	51.1 (11.6)	**.032**
	Control	67.9 (14.0)	55.2 (14.0)	** < .001**

Pre-existing represents the group of patients with a pre-existing THA and control represents the propensity score matched group of patients in the control group without a pre-existing THA. *p* values indicating pre- and postoperative differences in patients with pre-existing THA and the matched control group. Wilcoxon rank sum test was used and level of significance set at *p* < 0.05. SD = standard deviation. Significant differences are marked in bold

### Classification of pelvic mobility

The contribution of stiff pelvic mobility is distinctly smaller in the pre-existing THA group (25%) than in the control group (75%) preoperatively. The contribution of patients classified with stiff pelvic mobility decreased in both groups after THA. The contribution of patients with normal pelvic mobility preoperatively was larger in the pre-existing THA group (63.6%) than in the control group (22.7%) and increased in both groups after THA (Table 3).

**Table 3:** Contribution of pre- and postoperative pelvic mobility based on ∆ PT = PT_standing_ − PT_sitting_ defined as stiff (∆ PT < 10°), normal (∆ PT ≥ 10°–30°), and hypermobile (∆ PT ≥ 30°) according to the pre-existing THA group and the matched control group, % represents the percentage contribution; N represents the absolute number of patients

Classification of preoperative and postoperative pelvic mobility			
Pelvic mobility (∆ PT)		Pre-existing	Control
Stiff (%/*N*)	Preoperative	25.0 (11)	75.0 (33)
	Postoperative	9.1 (4)	18.2 (8)
Normal (%/*N*)	Preoperative	63.6 (28)	22.7 (10)
	Postoperative	68.2 (30)	75.0 (33)
Hypermobile (%/*N*)	Preoperative	11.4 (5)	2.3 (1)
	Postoperative	22.7 (10)	6.8 (3)

## Discussion

This study investigated the influence of a pre-existing THA compared to a propensity score matched control group without a pre-existing THA on the spinopelvic complex key parameters, lumbar flexibility, pelvic mobility and hip motion from prospectively collected data of patients undergoing elective primary THA. Both pre- and postoperative standing and relaxed sitting stereoradiographs were obtained to assess the effect of THA on the spinopelvic function and detect alterations in the classification of pelvic mobility. A considerable preoperative effort is made to identify patients with abnormal spinopelvic mobility, known to be at increased risk for THA dislocation; however the influence of frequent pre-existing THA on spinopelvic mobility is still unknown. It is important for arthroplasty surgeons to understand the complex relationship between the pelvis and the lumbar spine in order to identify high-risk patients and adapt the cup position in a dislocation-proof manner. The literature regarding the postoperative changes in spinopelvic mobility after THA is contradictory and inconsistent. On the one hand, variations in pelvic mobility are described [21, 22], while others describe no relevant alterations in pelvic mobility postoperatively [23–25].

It was demonstrated that the group with pre-existing THA had a significantly increased pelvic mobility compared to the control group preoperatively and postoperatively. Consequently, a threefold higher proportion of patients in the control group (75%) was classified as stiff regarding the pelvic mobility compared to patients with pre-existing THA (25%) preoperatively [19]. The classification of pelvic mobility revealed a clear shift in the contribution from stiffness to normal pelvic mobility after THA, especially in the control group without a pre-existing THA. The percentage of normal spinopelvic mobility in the control group increased postoperatively from 22.7 to 75%. However, even in the group with a pre-existing THA, the contribution of stiff pelvic mobility decreased from 25% to less than 10%. This effect can most likely be attributed to the recently performed THA and is associated with a release of muscle and capsular contractures. Another possible hypothesis for the significant alterations after THA might be the pain due to progressed osteoarthritis of the hip influencing the spinopelvic complex. As the source of pain was treated by the THA, postoperative adaption to a painless posture might take place, resulting in significant alterations of the spinopelvic complex. In our study, THA reduces pelvic retroversion in standing. Nevertheless, it should be critically noted that osteoarthritis of the hip is sometimes accompanied by a flexion contracture and thus pelvic anteversion. Following this theory, one might expect the opposite effect from THA, which was not observed in our investigation. Nevertheless, the THA-associated reduction in pelvic retroversion is worth mentioning, because it decreases the risk of posterior impingement and subsequent anterior THA dislocation in standing position. Accordingly, increased pelvic retroversion in standing position is reported as an associated factor for unfavorable pelvic mobility [26].

It may be assumed that THA not only affects pelvic mobility, but also lumbar flexibility. In comparison to the control group, the pre-existing group showed a significantly enhanced lumbar flexibility. We hypothesize THA increases the pelvic tilt in sitting position and decreases pelvic retroversion in standing position, leading to an increased pelvic mobility (∆ PT). In case of restricted pelvic mobility with inadequate backward tilting of the pelvis while sitting down, the lumbar lordosis in sitting position is compensatory increased to maintain upright upper body position. Due to postoperatively improved pelvic mobility the prior lumbar compensatory mechanism is no longer required, and physiological reduction in lumbar lordosis in sitting position is observed. Reduction in lumbar lordosis in sitting increases the difference between standing and sitting LL leading to significantly enhanced lumbar flexibility (∆ LL) postoperatively as observed in our investigation. This is of particular relevance as limited lumbar flexibility is considered a possible risk factor for THA dislocation [27] Furthermore, the influence of THA on the spinopelvic complex and in particular on the function of the lumbar spine is illustrated. The mutual effects between THA and the spine by spinopelvic interactions are also reflected in studies proving the reduction in low back pain after THA [25, 28].

The preoperatively increased hip motion (∆ PFA) in the control group is indicative of the known compensatory mechanism that a restriction in a specific segment of the spinopelvic complex is compensated by increased mobility in another segment [15]. Accordingly, preoperatively restricted pelvic mobility leads to compensatory increased hip motion within the spinopelvic complex. This mechanism is supported by our data with a postoperative increase in pelvic mobility in the control group and a subsequent reduction in hip motion. Which is of particular interest as increased hip motion is a known factor contributing to an increased risk of anterior impingement and subsequent posterior THA dislocation while sitting down [29, 30]. This leads to the assumption that THA has a protective influence on the dislocation risk through the postoperative reduction in compensatory increased hip motion.

From our point of view the crucial challenge is the preoperative identification of THA candidates with pathological spinopelvic mobility in order to adjust the acetabular cup position or to use a dual mobility cup. Consequently, there would be great clinical value in determining preoperatively individual factors which are associated with pathologic spinopelvic mobility. Accordingly, only the pre-selected patients with the highest odds for a pathologic spinopelvic mobility would be submitted to a radiological screening with standing and sitting radiographs causing enhanced radiation exposure and requiring a greater logistical and financial effort.

Our data suggest that patients without a pre-existing THA may have an increased risk for a postoperative THA dislocation due to restricted lumbar flexibility and pelvic mobility. However, the lack of a pre-existing THA is only one factor in the preoperative evaluation of spinopelvic mobility which might be considered. In addition, it is necessary to identify patients at risk, with a detailed history and physical examination. If a history of spinal fusion surgery, clinical postural sagittal imbalance, hip flexion contracture, or advanced arthritic alterations or surgical changes to the lumbosacral joint are noted on existing pelvic radiographs or in the clinical evaluation, the standardized radiological screening for spinopelvic pathologies with standing and sitting assessment should be considered. We do not derive a general recommendation to perform routine standing and sitting radiological screening for unilateral THA candidates without pre-existing THA from our study results.

We believe that our study contributes to the preoperative identification of patients at increased risk for pathologic spinopelvic mobility. Following our results, we identified a factor, which can support the arthroplasty surgeons in their evidence-guided decision-making process of which THA candidates should receive radiological screening for pathologic spinopelvic mobility preoperatively. Accordingly, our results may contribute to the avoidance of radiation exposure and decrease the financial burden of our healthcare system.

Some limitations of the study need to be addressed. Radiological assessments were performed during hospitalization and only short-term follow-up is presented, but long-term follow-up is planned. The immediate postoperative assessment was chosen because bony deformities of the pelvis and capsular contractures were relieved by THA and the position of the pelvis is mainly influenced by muscles. As we have chosen a short-term follow-up in expectation to detect direct effects of THA on the spinopelvic complex, subsequent alterations will not be detected. Although there is evidence that THA itself alters spinopelvic mobility, the underlying mechanisms remain hypothetical and require further research [20]. It might be raised critically that early postoperatively factors such as pain had an influence on the spinopelvic function. Due to the close monitoring of the patients' pain status and the application of an individual interdisciplinary pain protocol, which was developed in cooperation with the department of anesthesiology, and the minimally invasive surgical technique, we assume that the factor pain had a minor influence on the spinopelvic function. Nevertheless, we cannot exclude the influence of pain on the spinopelvic function by certainty. In conclusion, we are convinced that the short-term follow-up with significant changes in the spinopelvic complex after THA in a prospectively collected patient collective is valuable and important as a starting point for further investigations.

In our study, the relaxed seated position was selected as the functional assessment and a deep flexed seated or single leg standing position was not performed as an additional functional exercise. These functional images were not possible in the postoperative setting due to patient safety [10, 31, 32] We have chosen the standing position with hands on a support with relaxed upper limbs, because we intended to meet the patients' sense of safety after THA. The literature also describes a hand rest on the cheeks or collarbones as an alternative. This should be considered when interpreting the results. We do not assume a relevant influence of the severity of the osteoarthritis of the contralateral hip joint in the control group on spinopelvic mobility, since more than 72% (*N* = 32) of the patients in the control group presented only mild osteoarthritis (Kellgren and Lawrence grade 0–2) (Additional file 1). Nevertheless, the impact of severe osteoarthritis (Kellgren and Lawrence grade 3–4) on the spinopelvic mobility cannot be completely excluded.

In conclusion, a pre-existing THA was identified as an influencing factor on key parameter of spinopelvic mobility, namely pelvic mobility and lumbar flexibility. When trying to identify at risk populations of abnormal spinopelvic mobility and a correspondingly elevated risk of THA dislocation, our study indicates that pre-existing THA is a protective factor for pathological spinopelvic mobility. Therefore, our findings can serve to ensure that patients with pre-existing THA do not necessarily require additional diagnostics with standing and sitting lateral radiographs associated with radiation exposure and financial burden. In the preoperative spinopelvic mobility screening of THA patients, it might be focused on patients without a pre-existing THA. In addition, one should be aware that THA itself can alter key parameters of spinopelvic mobility.

## Supplementary Information

**Additional file 1. Supplement Table S1.** Interrater Reliability; **Supplement Table 2**. Description of the measured radiological spinopelvic Parameter

## Data Availability

The datasets generated and analyzed during the current study are available from the corresponding author on reasonable request.

## Abbreviations

THA: Total hip arthroplasty
BMI: Body mass index
LL: Lumbar lordosis
PT: Pelvic tilt
PFA: Pelvic femoral angle
∆ LL: Lumbar flexibility
∆ PT: Pelvic mobility
∆ PFA: Hip motion
PI: Pelvic incidence
PACS: Picture archiving and communication system
