# Supplementary material for: Spinopelvic mobility is influenced by pre-existing contralateral hip arthroplasty: a matched-pair analysis in patients undergoing hip replacement
Source: J Orthop Surg Res. 2022 Feb 2;17:64. doi: 10.1186/s13018-022-02945-5 (PMC8812232; doi:10.1186/s13018-022-02945-5)
Supplement: Supplementary file 1 — Additional file 1. Supplement Table S1. Interrater Reliability; Supplement Table 2. Description of the measured radiological spinopelvic Parameter [file 13018_2022_2945_MOESM1_ESM.docx]

## Supplements

**Interrater reliability**

|  | **Preoperative** | **Postoperative** | **Mean (Pre-postoperative)** | |  |
| --- | --- | --- | --- | --- | --- |
| Lumbar lordosis | .746 | .779 | | .763 | |
| Pelvic incidence | .534 | .728 | | .631 | |
| Pelvic tilt | .900 | .816 | | .858 | |
| Pelvic femoral angle | .694 | .831 | | .763 | |

**Supplement Table 1.** Pre-and postoperative values and the mean of interrater reliability of the global spinal alignment and spinopelvic parameters. Spearman´s rank correlation coefficient was used.

**Measured Radiological Parameter**

| **Radiological Parameter** | **Description** |
| --- | --- |
| Lumbar lordosis (LL) | Angle between superior endplate of L1 and superior endplate of S1. |
| Pelvic incidence (PI) | Angle between the line connecting the midpoint of the superior plate of S1 and the midpoint of the hip axis with the line perpendicular to the superior plate of S1. |
| Pelvic tilt (PT) | Angle between the line joining the midpoint of the hip axis to the midpoint of S1superior endplate and the vertical reference line |
| Pelvic femoral angle (PFA) | Angle between the center of the hip axis to the midpoint of the superior sacral endplate and a 10cm line from the center of the hip axis to the ventral cortex of the femur |

**Supplement Table 2**. Measured radiological parameters with description
